# Efficacy of acupuncture for recurrent implantation failure: a systematic review and meta-analysis of randomized controlled trials

**DOI:** 10.3389/fmed.2026.1758790

**Published:** 2026-04-14

**Authors:** Qin Huang, Xin-Yue Liu, Zi-Meng Zhu, Tian-Shu Hou, Yi Long, Si-Hui Li, Qian-Hui Huang, Hong-Mei Yang, Jun-Ran Wang, Qiao-Feng Wu

**Affiliations:** 1 Acupuncture and Moxibustion College, Chengdu University of Traditional Chinese Medicine, Chengdu, China; 2 Chengdu Integrated Traditional Chinese Medicine and Western Medicine Hospital, Chengdu, China; 3 Department of Traditional Chinese Medicine, Xinqiao Hospital, Third Military Medical University, Chongqing, China; 4 Pujiangxian Hospital of Traditional Chinese Medicine, Zhejiang, China

**Keywords:** acupuncture, embryo transfer, infertility, meta-analysis, recurrent implantation failure

## Abstract

**Background:**

Recurrent implantation failure (RIF) presents significant clinical and psychological challenges. While acupuncture is a potential adjunctive therapy during embryo transfer (ET), comprehensive evidence regarding its multidimensional effects remains limited. This study systematically evaluates acupuncture’s impact on pregnancy outcomes, endometrial receptivity, and psychological status in RIF patients.

**Methods:**

We performed a systematic review and meta-analysis of randomized controlled trials (RCTs) comparing acupuncture + ET with ET alone in patients with RIF. Eight major databases were searched from inception through October 2025. Risk of bias was assessed using the Risk of Bias 2 (RoB 2) tool, and the certainty of evidence was evaluated using the Grading of Recommendations Assessment, Development, and Evaluation (GRADE) framework.

**Results:**

Fourteen RCTs involving 1,428 patients were included. Regarding clinical efficacy, acupuncture + ET significantly increased the clinical pregnancy rate (relative risk [RR] = 1.73, 95% confidence interval [CI] [1.51, 1.99], *p* < 0.001), the embryo implantation rate (RR = 1.69, 95% CI [1.34, 2.13], *p* < 0.001), and the live birth rate (RR = 1.82, 95% CI [1.16, 2.86], *p* = 0.009), while reducing the miscarriage rate (RR = 0.40, 95% CI [0.17, 0.97], *p* = 0.04). In terms of endometrial receptivity, acupuncture significantly increased endometrial thickness (mean difference [MD] = 1.20, 95% CI [0.75, 1.66], *p* < 0.001) and reduced the endometrial pulsatility index (MD = −0.49, 95% CI [−0.93, −0.06], *p* = 0.03) and resistance index (MD = −0.21, 95% CI [−0.30, −0.13], *p* < 0.001). Furthermore, significant reductions in Self-Rating Anxiety Scale and Self-Rating Depression Scale scores were observed (MD = −5.89, *p* = 0.02 and MD = −6.83, *p* = 0.003, respectively). However, the certainty of evidence ranged from very low to moderate due to methodological limitations and high heterogeneity.

**Conclusion:**

Acupuncture may be a promising adjunctive therapy for RIF patients undergoing ET. However, the current evidence is constrained by significant methodological limitations. These results support the clinical integration of acupuncture into multidisciplinary RIF management but should be further confirmed by multicenter, rigorously blinded trials with standardized protocols and long-term outcome assessments.

**Systematic review registration:**

https://www.crd.york.ac.uk/prospero/display_record.php?RecordID=1237224, the Unique Identifier is PROSPERO CRD420251237224.

## 1 Introduction

Infertility remains a substantial public health issue worldwide, affecting approximately 15% of couples in their reproductive age (1). Embryo transfer (ET) has emerged as a pivotal intervention, offering a potential therapeutic option to many affected families. However, recurrent implantation failure (RIF) remains a major challenge, with approximately 10% of *in vitro* fertilization (IVF) patients experiencing this issue (2). RIF not only diminishes treatment success rates but also imposes substantial economic burdens and considerable psychological distress on patients (3). Specifically, women who experience multiple failed cycles often report elevated stress, underscoring the need for interventions that promote psychological well-being and treatment adherence (4). Currently, there is no widely accepted diagnostic standard for RIF. In clinical practice, it is often defined as the failure to establish a clinical pregnancy after two or three high-quality embryo transfers (5). Clinical outcomes in RIF are influenced by multiple factors, including age, embryo quality, body mass index (BMI), duration of infertility, and reproductive disorders such as endometrial polyps or submucosal fibroids (6–9). These factors affect endometrial receptivity, maternal immune function, and the overall likelihood of successful implantation, further complicating the management of RIF (10, 11). Given the multifactorial nature of RIF, no single treatment has been proven to be universally effective (12). Therefore, identifying effective treatment strategies remains a critical focus of current research.

Acupuncture, rooted in traditional Chinese medicine, has attracted increasing attention as a complementary treatment for RIF in assisted reproductive technology. Multiple studies have underscored its promising benefits, including improvements in clinical pregnancy rates, endocrine modulation, uterine perfusion, immune regulation, and anxiety reduction (13–17). While an earlier meta-analysis indicated that acupuncture might improve the clinical pregnancy rate, biochemical pregnancy rate, embryo implantation rate, and endometrial thickness (18), these conclusions warrant cautious interpretation. Limitations included a small number of eligible studies, variability in assisted reproductive protocols, and a wide range of outcome measures. Moreover, substantial heterogeneity across studies was not adequately addressed, and the overall certainty of evidence remains unclear.

Recent randomized controlled trials continue to investigate acupuncture as a treatment for RIF. To build on earlier findings and overcome prior methodological limitations, we conducted a comprehensive and rigorously designed systematic review and meta-analysis. This study evaluates whether acupuncture improves pregnancy outcomes, enhances endometrial receptivity, and supports psychological well-being in RIF patients undergoing embryo transfer. Through subgroup analyses and evidence grading with the Grading of Recommendations Assessment, Development and Evaluation (GRADE) system, we aim to provide reliable and clinically meaningful evidence regarding the adjunctive use of acupuncture for RIF.

## 2 Methods

This review was conducted in accordance with the Preferred Reporting Items for Systematic Reviews and Meta-Analyses (PRISMA) 2020 guidelines (Supplementary material). The study protocol was prospectively registered in the International Prospective Register of Systematic Reviews (PROSPERO) (Registration No. CRD420251237224).

### 2.1 Inclusion criteria

The inclusion criteria were as follows:

(1) Population: Patients diagnosed with RIF were those who had undergone at least 2–3 high-quality embryo transfers but had not achieved a clinical pregnancy (6), and were willing to continue with embryo transfer treatment.
(2) Intervention: Both groups underwent the same embryo transfer procedure. The treatment group (acupuncture + ET) received acupuncture therapy as an adjunct. Acupuncture interventions included manual acupuncture, electroacupuncture, transcutaneous electrical acupoint stimulation, auricular point, moxibustion, or a combination of acupuncture and moxibustion.
(3) Comparison: Participants in the control group underwent standard embryo transfer, with either sham acupuncture or no adjunctive intervention.
(4) Outcomes: Primary: clinical pregnancy rate; Secondary: embryo implantation rate, embryo transfer rate, biochemical pregnancy rate, live birth rate, miscarriage rate, endometrial thickness, endometrial pattern, endometrial pulsatility index, resistance index, Self-Rating Anxiety Scale scores, and Self-Rating Depression Scale scores.
(5) Study design: Randomized controlled trials (RCTs).

### 2.2 Exclusion criteria

The exclusion criteria were as follows:

(1) Non-randomized controlled trials, basic research, reviews, case reports, retrospective studies, dissertations, and conference abstracts;
(2) Studies with incomplete or missing data;
(3) Studies lacking primary outcome measures; and
(4) Studies without accessible full texts.

### 2.3 Search strategy

We searched eight databases through October 2025, including PubMed, Excerpta Medica Database (EMBASE), the Cochrane Library, China National Knowledge Infrastructure (CNKI), Chinese Scientific and Technical Periodicals Database (VIP), SinoMed, and Wanfang Data, for randomized controlled trials evaluating acupuncture as an adjunct to embryo transfer in patients with recurrent implantation failure. The search strategy incorporated relevant keywords and Medical Subject Headings (MeSH) terms related to “recurrent implantation failure,” “acupuncture,” and “randomized controlled trial” (Appendix 2). Additionally, we also screened the reference lists of pertinent publications, along with clinical guidelines, conference abstracts, and trial registries.

### 2.4 Literature screening and data extraction

Two reviewers independently performed study screening, data extraction, and quality assessment. Any disagreements were resolved through consultation with a third reviewer. Extracted data included study-level information and outcome measures. Study characteristics comprised the first author, year of publication, study design, sample size, mean age, duration of infertility, acupuncture modality, control intervention, adverse events, and reported outcomes. The outcome measures included clinical pregnancy rate, embryo implantation rate, embryo transfer rate, biochemical pregnancy rate, live birth rate, miscarriage rate, endometrial thickness, endometrial pattern, endometrial pulsatility index, resistance index, Self-Rating Anxiety Scale scores, and Self-Rating Depression Scale scores.

### 2.5 Certainty of evidence assessment

The certainty of evidence for each outcome was independently evaluated using the GRADE approach, which is widely recommended for systematic reviews of randomized controlled trials. This evaluation considered five domains: risk of bias, inconsistency, indirectness, imprecision, and publication bias.

### 2.6 Data synthesis and analysis

The meta-analysis was conducted using Review Manager 5.4. For continuous outcomes, mean differences (MDs) with 95% confidence intervals (CIs) were calculated, while dichotomous outcomes were expressed as risk ratios (RRs) with 95% CIs. Heterogeneity was measured using the chi-square test and the *I*^2^ statistic. A fixed-effects model was applied when the *p*-value was >0.10, and *I*^2^ was <50%. In contrast, a random-effects model was used if these criteria were not met. Sensitivity analyses were conducted by excluding individual studies to evaluate the robustness of the results. Subgroup analyses were performed when applicable, based on factors such as ET cycles in the RIF definition, fresh versus frozen ET, type of control, acupuncture intervention duration, total number of acupuncture sessions, and acupuncture type, to identify potential sources of heterogeneity. Publication bias was evaluated using funnel plots.

## 3 Results

### 3.1 Literature screening

**Figure 1:**
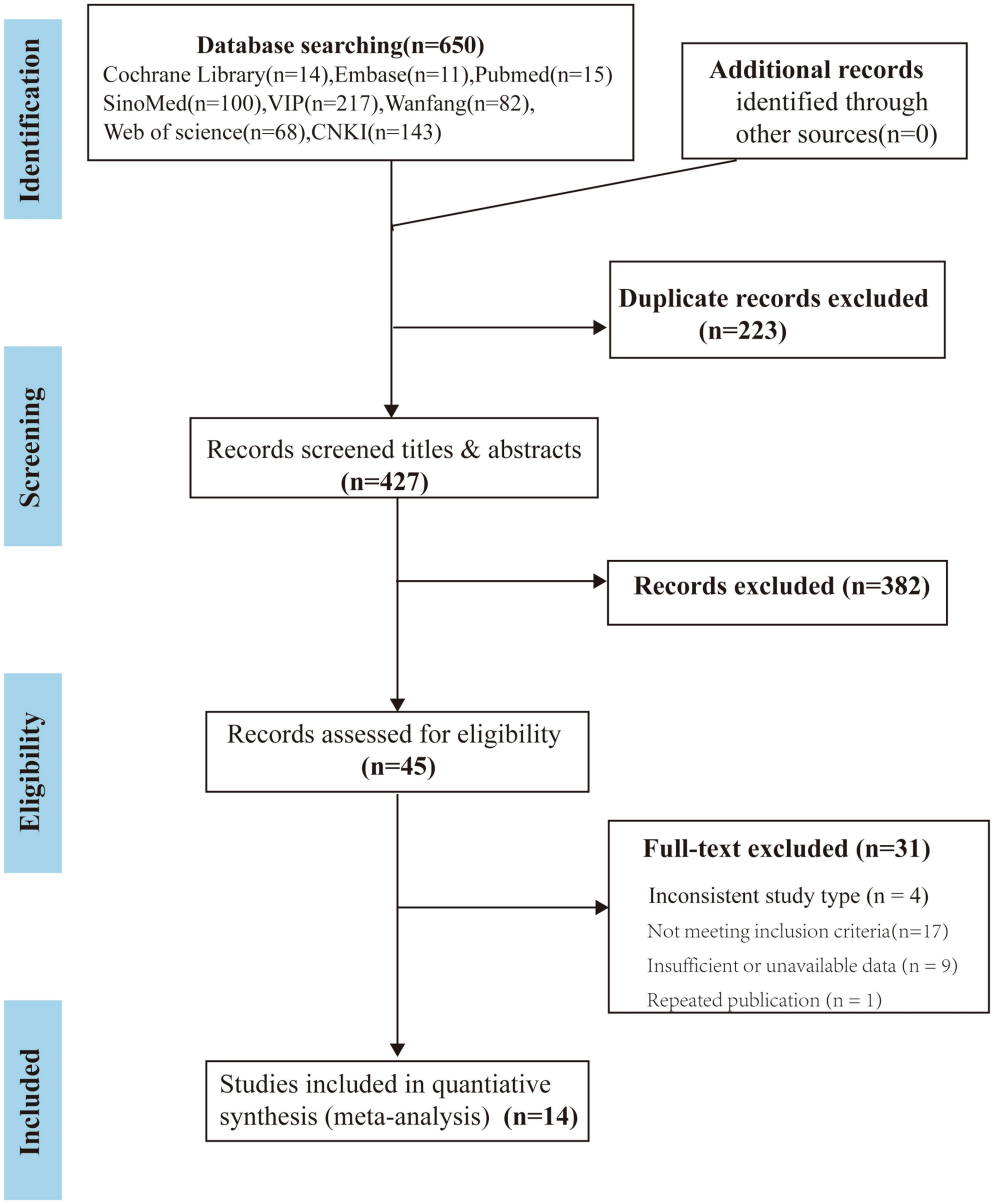
The PRISMA flowchart illustrating the study selection process.

We searched both Chinese and English databases, identifying 650 articles. After removing 223 duplicates, 427 records were screened by title and abstract, of which 382 were excluded for being irrelevant to the study topic or population. The full texts of the remaining 45 articles were assessed for eligibility. Thirty-one studies were excluded due to study type (*n* = 4), not meeting inclusion criteria (*n* = 17), insufficient or unavailable data (*n* = 9), or duplication (*n* = 1). Ultimately, 14 randomized controlled trials (19–32) were included in the meta-analysis. The study selection process is presented in the PRISMA flowchart (Figure 1).

### 3.2 Included study characteristics

This study included 14 randomized controlled trials with 1,428 RIF patients undergoing embryo transfer, comprising 709 in the acupuncture combined with ET group and 719 in the ET group. Sample sizes ranged from 28 to 108, with average ages between 30 and 36 years. Disease duration ranged from 3.96 to 5.1 years, although two studies did not report this information. Regarding interventions, the most frequent protocols were manual acupuncture alone (*n* = 4) and acupuncture combined with moxibustion (*n* = 3), followed by electroacupuncture (*n* = 3) and transcutaneous electrical acupoint stimulation (*n* = 2). Other modalities included auricular point pressing and warm needle moxibustion (*n* = 1 each). Intervention duration and frequency were reported in each study. The majority of patients in the control groups received no additional treatment, with only two studies using sham acupuncture. Two studies used fresh embryo transfer, eleven used frozen embryo transfer (FET), and one study did not describe whether it used fresh or frozen ET. The majority of studies reported multiple pregnancy and endometrial receptivity outcomes, with some also including anxiety and depression outcomes. Baseline differences (Table 1) indicate heterogeneity among the studies, necessitating subgroup and sensitivity analyses.

**Table 1:** Baseline characteristics of included studies.

Study	ET cycles for a diagnosis of RIF	Sample (T/C)	Age (T/C)	Disease duration (T/C)	Treatment	Control	Acupuncture intervention time and frequency	Outcomes
(1) Deng et al. (19)	≥3 times	45/46	35.2 ± 4.7/34.3 ± 5.8	NA	Auricular point + FET	FET	Pre- and post-transplant, 3 days, once daily	①⑦⑪⑫
(2) Ma et al. (20)	≥3 times	30/30	30 ± 3/31 ± 4	4.1 ± 1.9/4.7 ± 1.6	Needle-warming moxibustion + FET	FET	Daily once from M2 to pre-transplant	①③⑦⑧⑨⑩
(3) Li et al. (21)	>2 times	60/60	33.44 ± 6.61/33.52 ± 6.71	4.45 ± 2.13/4.38 ± 2.1	EA + FET	Sham EA + FET	Twice a week for 4 weeks prior to transplant	①②⑤⑥⑦⑧⑩
(4) Deng (22)	≥3times	49/51	35.52 ± 6.74/35.68 ± 7.25	4.75 ± 2.56/4.25 ± 3.15	EA + FET	FET	Every other day, from menstruation to pre-transplant	①④⑪⑫
(5) Cai et al. (23)	≥3 times	108/108	31.05 ± 5.33/30.46 ± 5.18	4.44 ± 0.58/4.36 ± 0.55	EA + FET	FET	Once every other day from M3 to pre- and post-transplant	①③
(6) Xu et al. (24)	≥3 times	82/94	32.5 ± 4.6/31.9 ± 4.3	5.1 ± 3.7/4.8 ± 3.6	TEAS + FET	FET	Daily once from M10 to pre- and post-transplant	①②⑤⑦
(7) Shuai et al. (25)	≥3 times	61/61	31.23 ± 3.78/31.58 ± 3.07	5.09 ± 2.78/5.71 ± 3.59	TEAS + fresh ET	Sham TEAS + Fresh ET	Daily once from D5 of ovulation induction cycle to pre-transplant	①②④⑦
(8) You et al. (26)	≥3 times	35/35	32.94 ± 1.79/33.29 ± 3.84	4.77 ± 2.99/5.06 ± 2.7	Acu + FET	FET	Three times a week for 3 menstrual cycles prior to transplant	①③④
(9) Ma and Zhang (27)	≥3 times	35/35	30.04 ± 2.98/30.55 ± 3.71	4.4 ± 1.8/4.9 ± 1.5	Acu + FET	FET	Once every other day from M2 to pre- and post-transplant	①③⑦⑧⑨⑩
(10) Li et al. (28)	≥3 times	45/40	34.46 ± 5.32/34.22 ± 4.64	NA	Acu + FET	FET	42 days prior to transplant, with each cycle lasting 14 days	①⑥⑦⑨⑩
(11) Villahermosa et al. (29)	>2 times	28/28	36 ± 2.7/36.4 ± 2.1	4.4 ± 1.5/4.7 ± 1.9	Acu&Mox + fresh ET	Fresh ET	Pre- and post-transplant: D1, D7, day before retrieval, and day 2 post-transplant (four times)	①⑦
(12) Xing et al. (30)	≥3 times	36/36	34 ± 2/34 ± 2	4.4 ± 1.1/4.4 ± 1.2	Acu&Mox + FET	FET	Three times a week for 3 months prior to transplant	①④⑥⑦⑧
(13) Xu et al. (31)	>2 times	45/45	33 ± 5/33 ± 5	3.96 ± 1.16/4.09 ± 1.15	Acu&Mox + FET	FET	Three times a week from M3 to pre-transplant	①⑤⑦⑧⑨⑩
(14) Zhao et al. (32)	>2 times	50/50	30 ± 3/31 ± 4	4.28 ± 1.31/4.75 ± 1.64	Acu&Mox + ET	ET	Once daily for 2 months prior to transplant	①③⑦⑨⑩⑪⑫

① Clinical pregnancy rate, ② embryo implantation rate, ③ embryo transfer rate, ④ live birth rate, ⑤ biochemical pregnancy rate, ⑥ miscarriage rate, ⑦ endometrial thickness, ⑧ endometrial pattern, ⑨ endometrial pulsatility index (PI), ⑩ resistance index (RI), ⑪ Self-Rating Anxiety Scale scores, and ⑫ Self-Rating Depression Scale scores.

T, treatment; C, control; TEAS, transcutaneous electrical acupoint stimulation; EA, electroacupuncture; Acu, acupuncture; Mox, moxibustion; M2/M3, the second/third day of the menstrual cycle.

### 3.3 Risk of bias assessment

**Figure 2:**
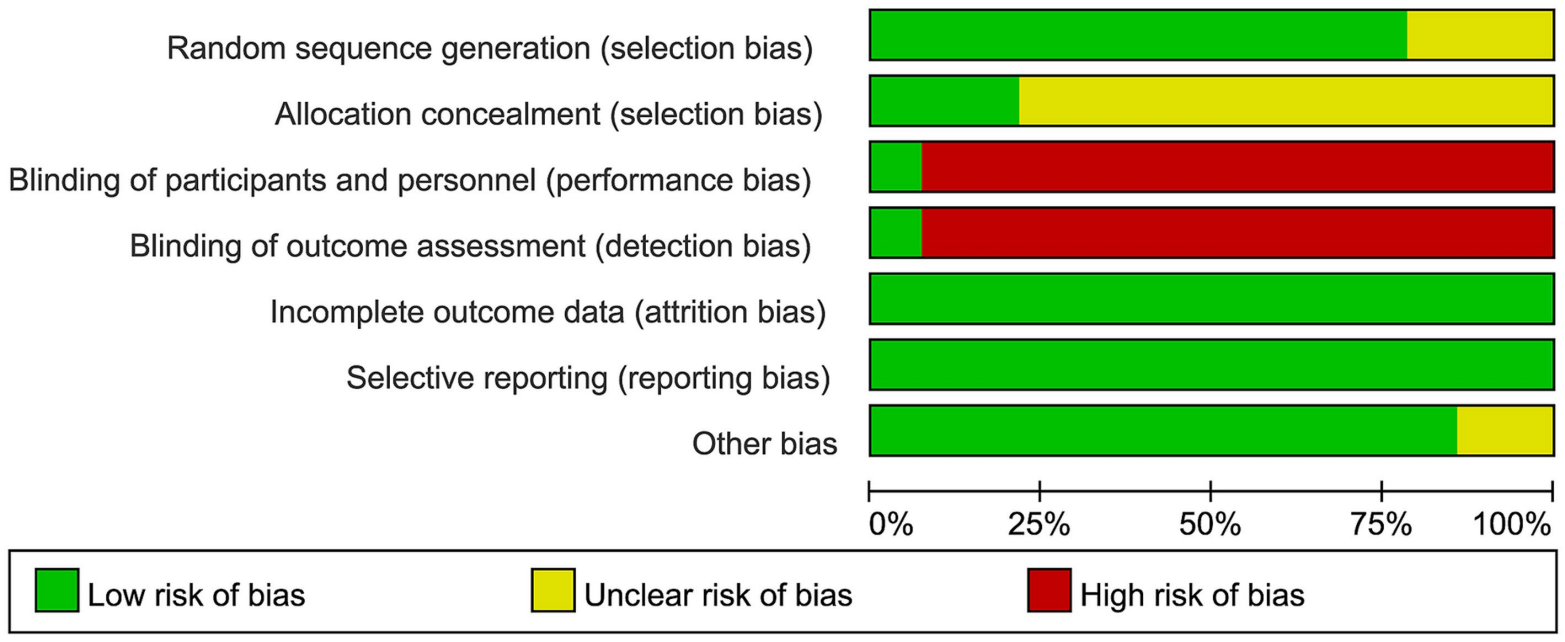
The risk of bias graph showing the distribution of low, unclear, and high risk across each domain for all studies.

**Figure 3:**
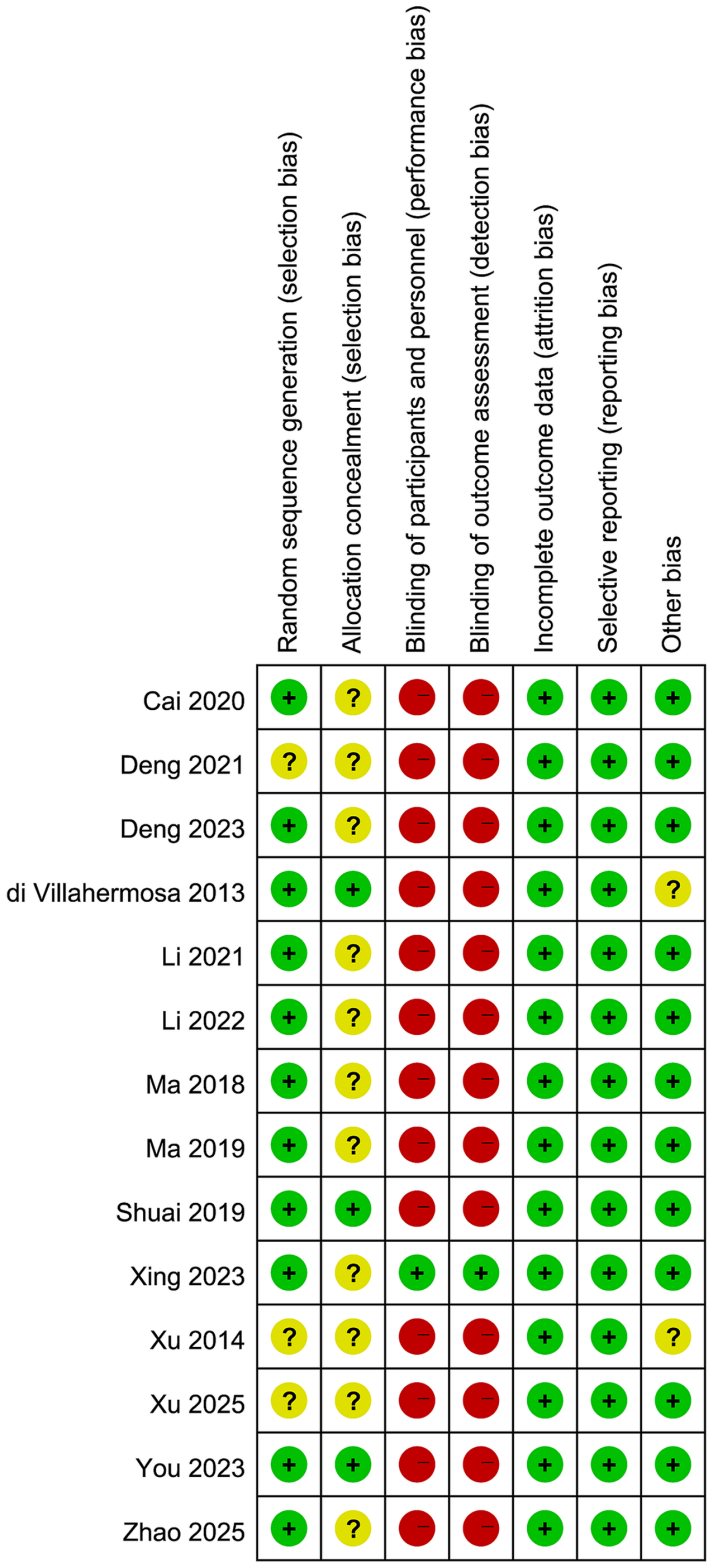
Risk of bias summary with green representing low risk, yellow representing unclear risk, and red representing high risk.

The risk of bias assessment indicated that 11 studies were rated as low risk for random sequence generation, while 3 studies were rated as unclear due to insufficient details. Regarding allocation concealment, 3 studies were rated as low risk, whereas 11 were unclear. The overall distribution of risk of bias across all methodological domains is illustrated in Figure 2. The majority of studies lacked blinding of participants, personnel, and outcome assessors, resulting in a high risk of bias, except for one study that was rated as low risk. All studies were considered low risk for incomplete outcome data and selective reporting. Only two studies were rated as unclear due to missing funding information, with the remainder rated as low risk. The detailed assessment of each individual study is shown in Figure 3.

### 3.4 Primary outcome

**Figure 4:**
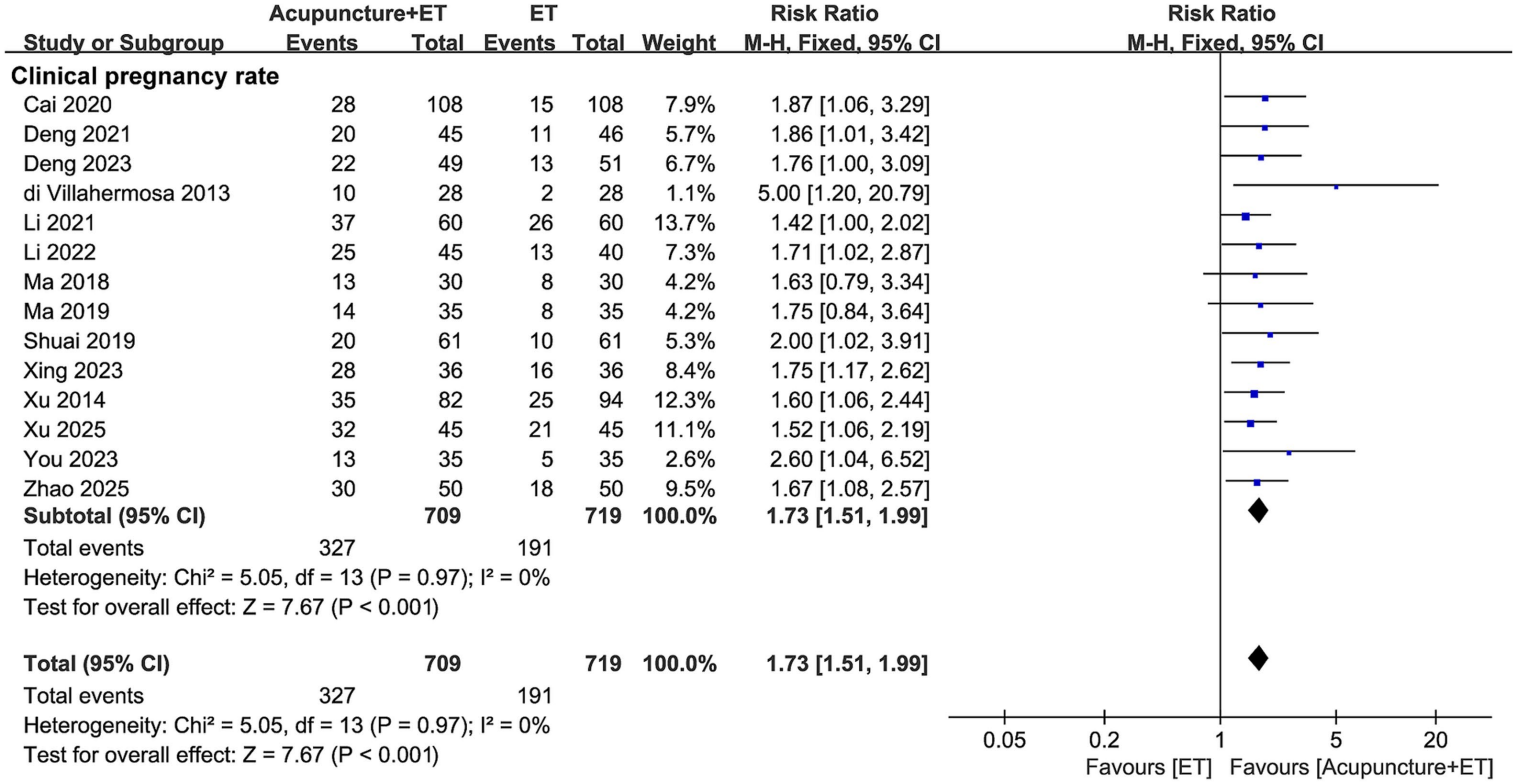
The forest plot comparing the clinical pregnancy rate between the acupuncture + ET group and the ET group.

All 14 studies reported clinical pregnancy rates, with low heterogeneity (*I*^2^ = 0%) (Figure 4). Using a fixed-effects model, acupuncture combined with embryo transfer significantly increased the clinical pregnancy rate compared with embryo transfer alone (RR = 1.73, 95% CI [1.51, 1.99], *p* < 0.001). These results indicate that acupuncture may improve clinical pregnancy outcomes in patients with RIF.

### 3.5 Secondary outcomes

#### 3.5.1 Pregnancy outcomes

**Figure 5:**
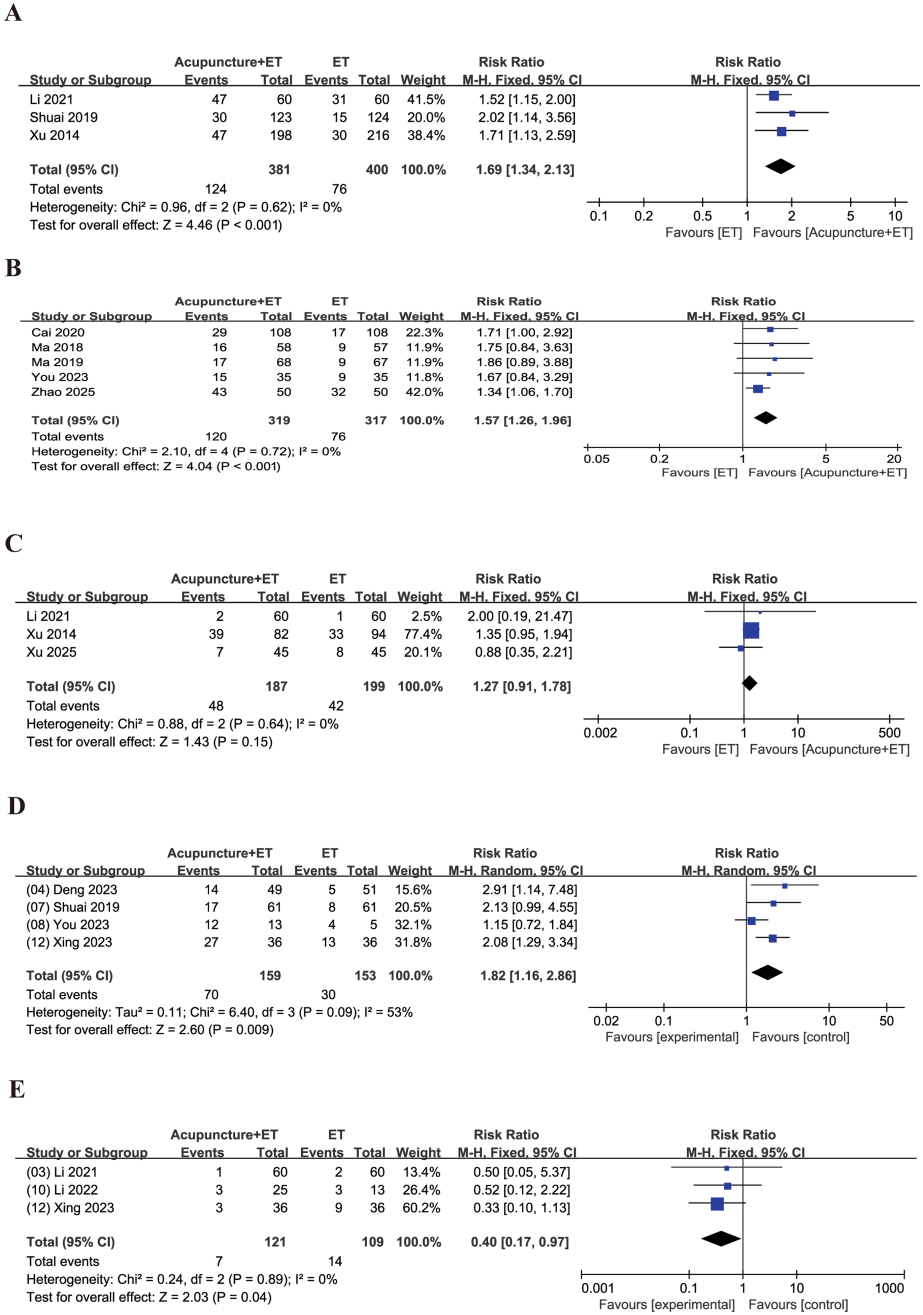
Forest plots comparing pregnancy outcomes between the acupuncture + ET group and the ET group. **(A)** Embryo implantation rate, **(B)** embryo transfer rate, **(C)** biochemical pregnancy rate, **(D)** live birth rate, and **(E)** miscarriage rate.

Compared with ET alone, acupuncture combined with ET alone significantly increased the embryo implantation rate (RR = 1.69, 95% CI [1.34, 2.13], *p* < 0.001, Figure 5A) and the embryo transfer rate (RR = 1.57, 95% CI [1.26, 1.96], *p* < 0.001, Figure 5B). No significant difference was observed in the biochemical pregnancy rate (RR = 1.27, 95% CI [0.91, 1.78], *p* = 0.15, Figure 5C). Combination therapy significantly increased the live birth rate (RR = 1.82, 95% CI [1.16, 2.86], *p* = 0.009, Figure 5D) and reduced the miscarriage rate (RR = 0.40, 95% CI [0.17, 0.97], *p* = 0.04, Figure 5E). Regarding statistical methods, only the live birth rate showed high heterogeneity (*I*^2^ = 53%) and was analyzed using a random-effects model. The other outcomes were analyzed using a fixed-effects model due to low heterogeneity. These findings indicate that acupuncture as an adjunct to ET may improve pregnancy outcomes in patients with RIF.

#### 3.5.2 Endometrial receptivity outcomes

**Figure 6:**
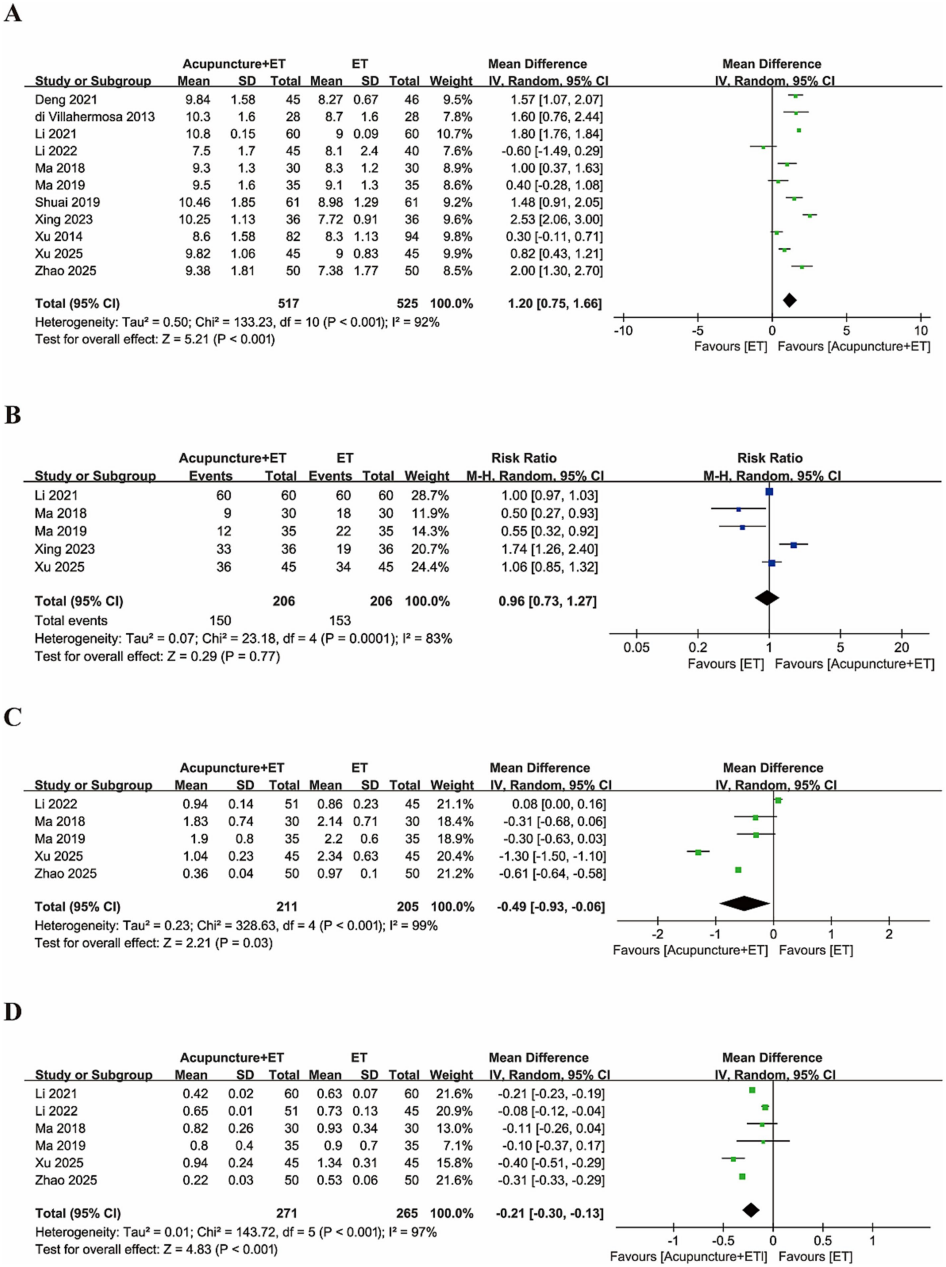
Forest plots comparing endometrial receptivity outcomes between the acupuncture + ET group and the ET group. **(A)** Endometrial thickness, **(B)** endometrial pattern, **(C)** endometrial pulse index, and **(D)** resistive index.

Acupuncture combined with ET significantly increased endometrial thickness (MD = 1.20, 95% CI [0.75, 1.66], *p* < 0.001, Figure 6A). No significant differences were observed in the endometrial pattern between the groups (RR = 0.96, 95% CI [0.73, 1.27], *p* = 0.77, Figure 6B). Combination therapy also led to significant reductions in the pulsatility index (MD = −0.49, 95% CI [−0.93, −0.06], *p* = 0.03) and the resistance index (MD = −0.21, 95% CI [−0.30, −0.13], *p* < 0.001), as shown in Figures 6C,D. A random-effects model was applied to all analyses because of the high heterogeneity observed in endometrial thickness (*I*^2^ = 92%), endometrial pattern (*I*^2^ = 83%), pulsatility index (*I*^2^ = 99%), and resistance index (*I*^2^ = 97%). Overall, these results indicate that acupuncture combined with embryo transfer may more effectively improve endometrial receptivity than ET alone in patients with RIF.

#### 3.5.3 Anxiety and depression outcomes

**Figure 7:**
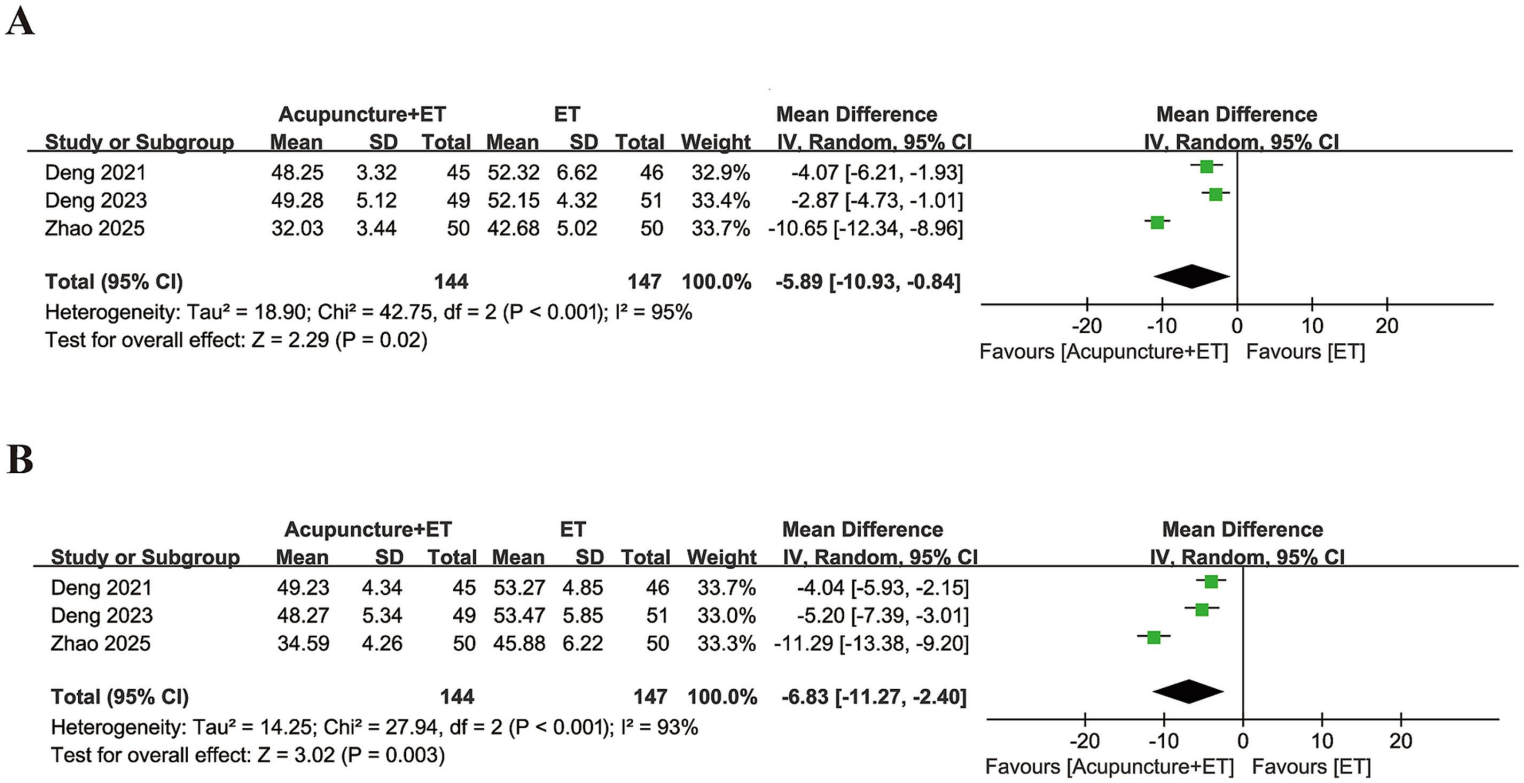
Forest plots comparing anxiety and depression outcomes between the acupuncture + ET group and the ET group. **(A)** Self-Rating Anxiety Scale and **(B)** Self-Rating Depression Scale.

Acupuncture combined with ET significantly reduced psychological distress compared with ET alone. Specifically, combination therapy led to significant decreases in Self-Rating Anxiety Scale scores (MD = −5.89, 95% CI [−10.93, −0.84], *p* = 0.02, Figure 7A) and Self-Rating Depression Scale scores (MD = −6.83, 95% CI [−11.27, −2.40], *p* = 0.003, Figure 7B). High heterogeneity was observed for both the anxiety scale (*I*^2^ = 95%) and the depression scale (*I^2^* = 93%); therefore, a random-effects model was applied to these analyses. These findings suggest that acupuncture may alleviate the psychological burden in patients with RIF, although the clinical interpretation should be approached with caution due to the limited number of included studies.

### 3.6 Subgroup analysis

Subgroup analyses were conducted to explore whether factors such as the number of ET cycles in the RIF definition, fresh versus frozen ET, control type, acupuncture intervention duration, total number of sessions, and acupuncture modality influenced the efficacy of acupuncture combined with embryo transfer (Appendix 3.1). Patients were first grouped based on the number of ET cycles. While combination therapy significantly improved overall clinical pregnancy rates, the effect was more pronounced in patients with three or more ET cycles, suggesting that acupuncture may be particularly beneficial for those with a longer history of implantation failure. Outcomes were then analyzed according to fresh versus frozen ET. Fresh ET showed greater improvements in clinical pregnancy rates compared with frozen ET, but only two studies evaluated fresh ET, highlighting the urgent need for further high-quality RCTs.

Subgroup analyses based on the presence of sham acupuncture in the control group indicated that the clinical pregnancy rate was higher when no sham acupuncture was used, suggesting that sham interventions may attenuate some of the benefits of acupuncture. The influence of the acupuncture technique was also examined. The majority of techniques improved clinical pregnancy rates, whereas needle-warming moxibustion did not produce significant effects. Excluding the single study on needle-warming moxibustion did not change the overall effect size (RR = 1.78, 95% CI [1.53, 2.07], *p* < 0.001), and the main conclusion remained consistent (RR = 1.73, 95% CI [1.51, 1.99], *p* < 0.001) (Appendix 3.2), indicating the robustness of the results despite technique heterogeneity. Finally, analyses of interventions administered before or after embryo transfer, along with those with more than 10 sessions, showed greater improvements in the clinical pregnancy rate, indicating that long-term and regular acupuncture treatments may enhance therapeutic outcomes.

### 3.7 Sensitivity analysis, publication bias, and certainty of evidence

We performed a sensitivity analysis of the primary outcome, the clinical pregnancy rate. A leave-one-out analysis showed that excluding individual studies did not substantially change the effect size or direction of the results, confirming the stability of our findings. Additionally, a methodological sensitivity analysis was conducted by removing three low-quality studies that lacked adequate randomization details or blinding. A reanalysis of the remaining 11 studies using a fixed-effects model yielded a combined effect size of RR = 1.78 (95% CI [1.50, 2.10], *p* < 0.001), consistent with the original analysis (RR = 1.73, 95% CI [1.51, 1.99], *p* < 0.001), supporting the robustness of the conclusions (Appendix 4).

**Figure 8:**
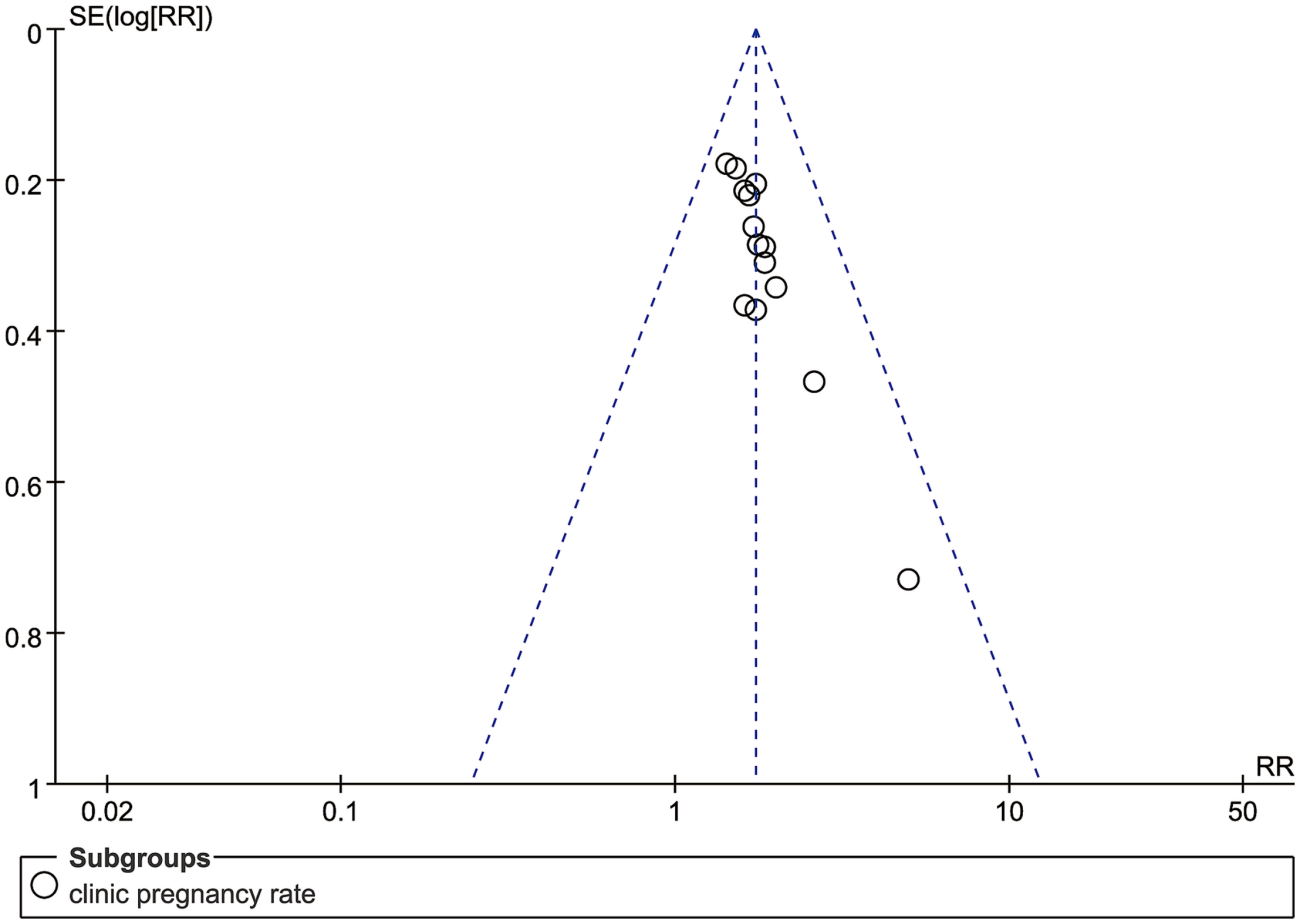
The funnel plot assessing publication bias for the clinical pregnancy rate.

Inspection of the funnel plot showed that the effect sizes for clinical pregnancy rates were symmetrically distributed, indicating the absence of substantial publication bias (Figure 8). Confidence in the evidence for all outcomes was assessed using the GRADE approach. The majority of outcomes were rated as low or very low, with only a few rated as moderate, primarily due to risks of bias and heterogeneity. These findings indicate that, although the statistical results are robust, the clinical interpretation should be approached with caution (Appendix 5).

### 3.8 Safety of intervention

Only two studies reported adverse events. Minor issues, such as itching and fainting, were noted in one study, all of which resolved with treatment (22). Another study (30) found no significant difference in adverse events, without linking them to acupuncture. The remaining studies did not report on intervention safety.

## 4 Discussion

This study represents the most recent and comprehensive systematic review and meta-analysis assessing the efficacy of acupuncture for recurrent implantation failure. By screening data from 14 randomized controlled trials involving 1,428 patients, we demonstrated that acupuncture serves as a holistic intervention yielding multidimensional benefits. Our findings indicate that acupuncture combined with embryo transfer significantly improves a broad range of pregnancy outcomes, including the clinical pregnancy rate, embryo implantation rate, and live birth rate. Specifically, the results show a significant increase in implantation and live birth rates, alongside a reduction in the miscarriage rate. The therapy enhances endometrial receptivity and alleviates psychological distress. Although the quality of evidence ranged from very low to moderate due to risk of bias and heterogeneity, the data consistently indicate that acupuncture is a beneficial adjunctive therapy. However, safety data remain limited as adverse events were sparsely reported across studies, highlighting the need for uniform monitoring in future investigations.

The interpretation of clinical efficacy is contingent upon both the selection of outcome indicators and the specific design of control groups. Standard evaluation of pregnancy outcomes includes key indicators such as clinical pregnancy, embryo implantation, live birth, and miscarriage rates (33). While previous reviews focused primarily on biochemical markers, our study emphasizes the live birth rate as a more definitive measure of clinical utility. Notably, although earlier studies reported improvements in biochemical pregnancy rates (18), our analysis did not confirm this finding, likely reflecting the enhanced methodological rigor and larger sample sizes of the more recent trials included in our review. Furthermore, subgroup analyses provided critical insights into treatment protocols. We found that patients with a longer history of RIF and those undergoing fresh embryo transfer cycles derived greater benefit. However, the presence of sham acupuncture in control groups appeared to attenuate the estimated treatment effect. This observation underscores a critical methodological challenge: Sham interventions are often not physiologically inert (34, 35). Sham acupuncture, which typically involves non-penetrating needles or needling at non-acupoints, may still elicit physiological responses through cutaneous stimulation. Such stimulation can modulate the nervous system, thereby attenuating the statistical differences between groups. Regarding specific techniques, the majority of modalities improved outcomes, with the exception of needle-warming moxibustion. Importantly, excluding the single study utilizing this technique did not alter the overall effect size, confirming the robustness of our main conclusion despite the heterogeneity in treatment methods. Finally, a distinct dose–response relationship was observed: interventions administered both before and after embryo transfer, and those exceeding 10 sessions, yielded superior improvements. This suggests that long-term, regular acupuncture is necessary, and that a cumulative dose may be required to induce the neuroendocrine changes essential for treatment success.

The weakened fertilization ability of the endometrium is the primary cause of embryo implantation failure, accounting for approximately two-thirds of the failure cases (36, 37). Endometrial receptivity is typically evaluated by thickness, morphology, and uterine blood flow (38). The present analysis demonstrates that acupuncture effectively reverses non-receptive states by significantly increasing endometrial thickness and optimizing uterine perfusion, as evidenced by reduced pulsatility and resistance indices (39). These findings suggest the observed clinical benefits are likely attributable to the optimization of this key factor. Physiologically, such a reduction in arterial impedance enhances the delivery of oxygen and nutrients essential for endometrial proliferation. Although significant changes in endometrial morphological patterns were not observed, the hemodynamic improvements indicate functional restoration of the uterine environment. These effects are likely mediated by upstream neuroendocrine alterations, specifically in the regulation of reproductive hormones. Previous studies have demonstrated that acupuncture can restore the balance of estrogen and progesterone and upregulate their receptors, thereby enhancing endometrial receptivity, correcting displacement of the implantation window, and ultimately increasing pregnancy rates (40). Additionally, endometrial receptivity is intricately linked to the immune microenvironment. Acupuncture has been shown to modulate immune cell subsets, restore the Th2/Th1 balance, and increase the levels of leukemia inhibitory factor (LIF) and IL-12, thereby regulating the local immune-inflammatory milieu, enhancing endometrial receptivity, and promoting embryo implantation (41).

Current evidence indicates that patients with RIF are particularly prone to anxiety and depression (42, 43). Such psychological stress may impair embryo implantation by inducing endocrine dysregulation and increasing uterine contractions (44). Consequently, addressing the psychological state of RIF patients is paramount. Our findings corroborate previous evidence that acupuncture effectively alleviates these symptoms. Compared with embryo transfer alone, the acupuncture-combined group demonstrated significantly reduced anxiety and depression levels. However, given the limited sample size, these results should be interpreted with caution, underscoring the need for further robust research.

Despite these promising findings, several limitations warrant caution. First, although this is the largest meta-analysis to date, the geographic concentration of the studies in China limits the generalizability of the results to other populations. Second, substantial heterogeneity persisted across studies, largely due to variations in RIF definitions and embryo quality. Third, incomplete reporting of acupuncture parameters, particularly needle depth, precluded a precise dose–response analysis. Finally, methodological shortcomings in allocation concealment and blinding, coupled with limited adverse event reporting, constrain the overall strength of the evidence.

To address these knowledge gaps, future research should prioritize large-scale, multicenter randomized controlled trials across diverse geographic regions. Standardization of inclusion criteria and acupuncture protocols is essential to minimize heterogeneity. Moreover, researchers should rigorously implement blinding procedures and systematically collect detailed clinical data to identify key covariates affecting efficacy. Furthermore, comprehensive reporting of adverse events and long-term outcomes, including live birth rates and quality of life, is necessary to validate the safety and sustained clinical value of acupuncture.

## 5 Conclusion

Based on this comprehensive meta-analysis, acupuncture appears to be a promising adjunctive therapy for RIF patients undergoing embryo transfer. The intervention demonstrates multidimensional therapeutic benefits, including significant improvements in clinical pregnancy and live birth rates, reductions in miscarriage rates, enhanced endometrial receptivity, and alleviation of anxiety and depressive symptoms. However, the certainty of evidence supporting these findings ranges from very low to moderate, primarily due to the risk of bias, substantial heterogeneity, and the potential confounding effects of sham control designs. Consequently, these results should be interpreted cautiously in clinical practice. Future research should focus on high-quality, multicenter RCTs with rigorous blinding and standardized protocols, along with comprehensive monitoring of adverse events and long-term follow-up, to establish the safety profile and sustained efficacy of acupuncture in reproductive medicine.

## Data Availability

The original contributions presented in the study are included in the article/Supplementary material, further inquiries can be directed to the corresponding author.
