# Supplementary material for: Efficacy of acupuncture for recurrent implantation failure: a systematic review and meta-analysis of randomized controlled trials
Source: Front Med (Lausanne). 2026 Apr 14;13:1758790. doi: 10.3389/fmed.2026.1758790 (PMC13121254; doi:10.3389/fmed.2026.1758790)
Supplement: Supplementary file 1 [file Data_Sheet_1.pdf]

Supplementary Material

1 Appendix 1: PRISMA Checklist.

Section and Topic	Item #	Checklist item	Location where item is reported
**TITLE**			
Title	1	Identify the report as a systematic review.	P 1
**ABSTRACT**			
Abstract	2	See the PRISMA 2020 for Abstracts checklist.	P 3
**INTRODUCTION**			
Rationale	3	Describe the rationale for the review in the context of existing knowledge.	P 2
Objectives	4	Provide an explicit statement of the objective(s) or question(s) the review addresses.	P 3
**METHODS**			
Eligibility criteria	5	Specify the inclusion and exclusion criteria for the review and how studies were grouped for the syntheses.	P 4
Information sources	6	Specify all databases, registers, websites, organisations, reference lists and other sources searched or consulted to identify studies. Specify the date when each source was last searched or consulted.	P 4
Search strategy	7	Present the full search strategies for all databases, registers and websites, including any filters and limits used.	P5 & Appendix 1
Selection process	8	Specify the methods used to decide whether a study met the inclusion criteria of the review, including how many reviewers screened each record and each report retrieved, whether they worked independently, and if applicable, details of automation tools used in the process.	P 5
Data collection process	9	Specify the methods used to collect data from reports, including how many reviewers collected data from each report, whether they worked independently, any processes for obtaining or confirming data from study investigators, and if applicable, details of automation tools used in the process.	P 5
Data items	10a	List and define all outcomes for which data were sought. Specify whether all results that were compatible with each outcome domain in each study were sought (e.g. for all measures, time points, analyses), and if not, the methods used to decide which results to collect.	P 5
	10b	List and define all other variables for which data were sought (e.g. participant and intervention characteristics, funding sources). Describe any assumptions made about any missing or unclear information.	P 5
Study risk of bias assessment	11	Specify the methods used to assess risk of bias in the included studies, including details of the tool(s) used, how many reviewers assessed each study and whether they worked independently, and if applicable, details of automation tools used in the process.	P 5
Effect measures	12	Specify for each outcome the effect measure(s) (e.g. risk ratio, mean difference) used in the synthesis or presentation of results.	P 5
Synthesis methods	13a	Describe the processes used to decide which studies were eligible for each synthesis (e.g. tabulating the study intervention characteristics and comparing against the planned groups for each synthesis (item #5)).	P 6
	13b	Describe any methods required to prepare the data for presentation or synthesis, such as handling of missing summary statistics, or data conversions.	P 6

Section and Topic	Item #	Checklist item	Location where item is reported
	13c	Describe any methods used to tabulate or visually display results of individual studies and syntheses.	P 6
	13d	Describe any methods used to synthesize results and provide a rationale for the choice(s). If meta-analysis was performed, describe the model(s), method(s) to identify the presence and extent of statistical heterogeneity, and software package(s) used.	P 6
	13e	Describe any methods used to explore possible causes of heterogeneity among study results (e.g. subgroup analysis, meta-regression).	P 6
	13f	Describe any sensitivity analyses conducted to assess robustness of the synthesized results.	P 6
Reporting bias assessment	14	Describe any methods used to assess risk of bias due to missing results in a synthesis (arising from reporting biases).	P 6
Certainty assessment	15	Describe any methods used to assess certainty (or confidence) in the body of evidence for an outcome.	P 5
**RESULTS**			
Study selection	16a	Describe the results of the search and selection process, from the number of records identified in the search to the number of studies included in the review, ideally using a flow diagram.	P 6
	16b	Cite studies that might appear to meet the inclusion criteria, but which were excluded, and explain why they were excluded.	P 6
Study characteristics	17	Cite each included study and present its characteristics.	P 6
Risk of bias in studies	18	Present assessments of risk of bias for each included study.	P 6
Results of individual studies	19	For all outcomes, present, for each study: (a) summary statistics for each group (where appropriate) and (b) an effect estimate and its precision (e.g. confidence/credible interval), ideally using structured tables or plots.	P 7
Results of syntheses	20a	For each synthesis, briefly summarise the characteristics and risk of bias among contributing studies.	P 7
	20b	Present results of all statistical syntheses conducted. If meta-analysis was done, present for each the summary estimate and its precision (e.g. confidence/credible interval) and measures of statistical heterogeneity. If comparing groups, describe the direction of the effect.	P 7
	20c	Present results of all investigations of possible causes of heterogeneity among study results.	P 8
	20d	Present results of all sensitivity analyses conducted to assess the robustness of the synthesized results.	P 9
Reporting biases	21	Present assessments of risk of bias due to missing results (arising from reporting biases) for each synthesis assessed.	P 9
Certainty of evidence	22	Present assessments of certainty (or confidence) in the body of evidence for each outcome assessed.	P 9
**DISCUSSION**			
Discussion	23a	Provide a general interpretation of the results in the context of other evidence.	P 9
	23b	Discuss any limitations of the evidence included in the review.	P10
	23c	Discuss any limitations of the review processes used.	P10
	23d	Discuss implications of the results for practice, policy, and future research.	P10
**OTHER INFORMATION**			

Section and Topic	Item #	Checklist item	Location where item is reported
Registration and protocol	24a	Provide registration information for the review, including register name and registration number, or state that the review was not registered.	P 3
	24b	Indicate where the review protocol can be accessed, or state that a protocol was not prepared.	P 3
	24c	Describe and explain any amendments to information provided at registration or in the protocol.	none
Support	25	Describe sources of financial or non-financial support for the review, and the role of the funders or sponsors in the review.	P13
Competing interests	26	Declare any competing interests of review authors.	P13
Availability of data, code and other materials	27	Report which of the following are publicly available and where they can be found: template data collection forms; data extracted from included studies; data used for all analyses; analytic code; any other materials used in the review.	P13

2 Appendix 2: Search strategy

Search strategy of PubMed、EMBASE、Cochrane、Web of science

#	Searches
1	((((((((Recurrent Implantation Failure[Title/Abstract]) OR (RIF[Title/Abstract])) OR (repeated IVF-ET failure[Title/Abstract])) OR (repeated implantation failure[Title/Abstract])) OR ((repeated failure of implantation[Title/Abstract])) OR (recurrent IVF-ET failures[Title/Abstract])) OR (recurrent embryo implantation failure[Title/Abstract])) OR (repeated in vitro fertilization[Title/Abstract] OR embryo transfer failure[Title/Abstract]))
2	((((((((((((((((((((((Acupuncture[MeSH Terms]) OR (Pharmacopuncture[Title/Abstract])) OR (Acupuncture Therapy[Title/Abstract])) OR (Acupuncture Points[Title/Abstract])) OR (Acupuncture Analgesia[Title/Abstract])) OR (Electroacupuncture[Title/Abstract])) OR (Moxibustion[Title/Abstract])) OR (Acustimulation[Title/Abstract])) OR (electro-acupuncture[Title/Abstract])) OR (Needling[Title/Abstract])) OR (needle pricking[Title/Abstract])) OR (fire needle[Title/Abstract])) OR (point application[Title/Abstract])) OR (three edged needle[Title/Abstract])) OR (auricular therapy[Title/Abstract])) OR (point therapy[Title/Abstract])) OR (moxabustion[Title/Abstract])) OR (transcutaneous electrical acupoint stimulation[Title/Abstract])) OR (TEAS[Title/Abstract])) OR (point injection[Title/Abstract])) OR (acupoint embedding[Title/Abstract])) OR (acupoint catgut embedding[Title/Abstract]))) OR (Warming needle moxibustion[Title/Abstract])
3	(((((((randomized controlled trial[Title/Abstract]) OR (randomised controlled trial[Title/Abstract])) OR (Controlled Clinical Trial[Title/Abstract])) OR (Randomised[Title/Abstract])) OR (Randomized[Title/Abstract])) OR (Randomly[Title/Abstract])) OR (Trial[Title/Abstract])) OR (RCT[Title/Abstract])
4	#1 AND #2 AND #3

Search strategy of CNKI、SinoMed、VIP、Wanfang

#	Searches
1	反复种植失败 OR 重复着床失败 OR 反复着床失败 OR 胚胎反复移植失败 OR 反复移植失败 OR RIF OR 辅助生殖技术失败 OR 体外受精胚胎移植失败 OR IVF-ET 失败
2	针灸 OR 针刺 OR 电针 OR 经皮电刺激 OR 艾灸 OR 温针灸 OR 耳穴 OR 穴位埋线 OR 穴位注射 OR 火针
3	随机对照试验 OR 临床试验 OR 随机 OR 对照 OR 试验 OR 分组
4	#1 AND #2 AND #3

3 Appendix 3: Subgroup analysis

3.1 Table of subgroup analysis

Outcome	Number of studies	Heterogeneity		Meta analysis		Subgroup differences
		P value	I^2	Effect estimate(95%CI)	*P* value	
**Meta-analysis results by clinical pregnancy rate**						
ET cycles in the RIF definition	14	0.97	0%	1.73(1.51,1.99)	< 0.001*	0.51
> 2 times	4	0.38	3%	1.63(1.31,2.02)	< 0.001*	
≥ 3 times	10	1.00	0%	1.79(1.49,2.14)	< 0.001*	
fresh/frozen ET	13	0.97	0%	1.74(1.50,2.02)	< 0.001*	0.21
fresh ET	2	0.25	25%	2.5(1.37,4.57)	0.003*	
frozen ET	11	0.99	0%	1.68(1.44,1.96)	< 0.001*	
type of control	14	0.97	0%	1.75(1.52,2.03)	< 0.001*	0.49
Sham	2	0.37	0%	1.58(1.15,2.18)	0.005*	

No additional treatment	12	0.97	0%	1.80(1.53,2.11)	< 0.001*	
type of acupuncture	14	0.97	0%	1.73(1.51,1.99)	< 0.001*	0.99
acupuncture	3	0.72	0%	1.89(1.28,2.78)	0.001*	
auricular point	1	-	-	1.86(1.01,3.42)	0.05*	
needle warming moxibustion	1	-	-	1.63(0.79,3.34)	0.19	
EA	3	0.65	0%	1.63(1.24,2.13)	0.0004*	
Acu&Mox	4	0.44	0%	1.75(1.39,2.21)	< 0.001*	
TEAS	2	0.58	0%	1.72(1.21,2.46)	0.003*	
acupuncture intervention duration	14	0.97	0%	1.73(1.51,1.99)	< 0.001*	0.54
Pre-transplant	9	0.97	0%	1.68(1.43,1.97)	< 0.001*	
Pre- and post-transplant	5	0.67	0%	1.85(1.41,2.42)	< 0.001*	
total number of acupuncture sessions	14	0.97	0%	1.73(1.51,1.99)	< 0.001*	0.95
> 10 times	9	0.99	0%	1.74(1.47,2.06)	< 0.001*	
≤ 10 times	5	0.50	0%	1.72(1.34,3.21)	< 0.001*	

3.2 Excluded needle warming moxibustion

Study or Subgroup	Acupuncture+ET Events	Acupuncture+ET Total	ET Events	ET Total	Weight	Risk Ratio M-H, Fixed, 95% CI
Cai 2020	28	108	15	108	9.2%	1.87 [1.06, 3.29]
Deng 2021	20	45	11	46	6.6%	1.86 [1.01, 3.42]
Deng 2023	22	49	13	51	7.8%	1.76 [1.00, 3.09]
di Villahermosa 2013	10	28	2	28	1.2%	5.00 [1.20, 20.79]
Li 2022	25	45	13	40	8.4%	1.71 [1.02, 2.87]
Ma 2018	13	30	8	30	4.9%	1.63 [0.79, 3.34]
Ma 2019	14	35	8	35	4.9%	1.75 [0.84, 3.64]
Shuai 2019	20	61	10	61	6.1%	2.00 [1.02, 3.91]
Xing 2023	28	36	16	36	9.8%	1.75 [1.17, 2.62]
Xu 2014	35	82	25	94	14.2%	1.60 [1.06, 2.44]
Xu 2025	32	45	21	45	12.8%	1.52 [1.06, 2.19]
You 2023	13	35	5	35	3.1%	2.60 [1.04, 6.52]
Zhao 2025	30	50	18	50	11.0%	1.67 [1.08, 2.57]
**Total (95% CI)**		**649**		**659**	**100.0%**	**1.78 [1.53, 2.07]**
Total events	290		165			

Heterogeneity: Chi² = 3.96, df = 12 (P = 0.98); I² = 0%

Test for overall effect: Z = 7.42 (P < 0.001)

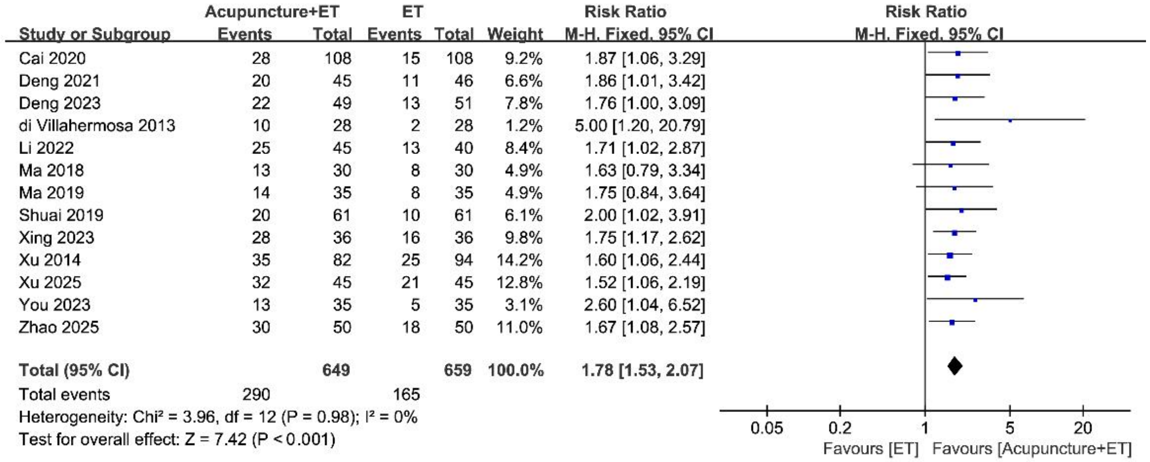

4 Appendix 4: Sensitivity analysis：Clinical pregnancy rate

4.1

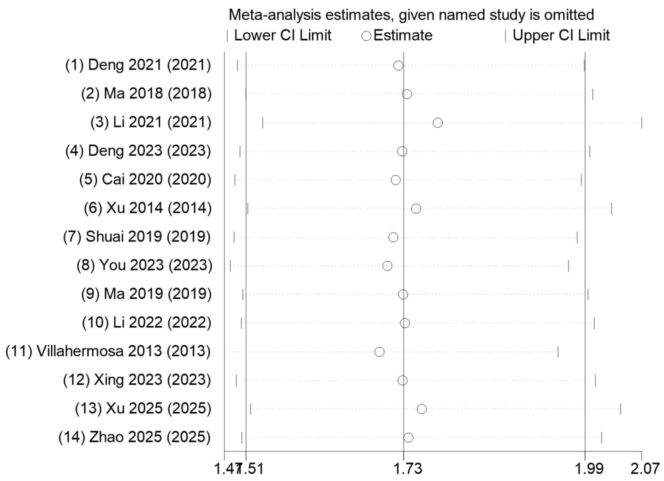

4.1 Excluded low-quality studies

Study or Subgroup	Acupuncture+ET Events	Acupuncture+ET Total	ET Events	ET Total	Weight	Risk Ratio M-H, Fixed, 95% CI
Cai 2020	28	108	15	108	11.2%	1.87 [1.06, 3.29]
Deng 2023	22	49	13	51	9.5%	1.76 [1.00, 3.09]
di Villahermosa 2013	10	28	2	28	1.5%	5.00 [1.20, 20.79]
Li 2021	37	60	26	60	19.3%	1.42 [1.00, 2.02]
Li 2022	25	45	13	40	10.2%	1.71 [1.02, 2.87]
Ma 2018	13	30	8	30	5.9%	1.63 [0.79, 3.34]
Ma 2019	14	35	8	35	5.9%	1.75 [0.84, 3.64]
Shuai 2019	20	61	10	61	7.4%	2.00 [1.02, 3.91]
Xing 2023	28	36	16	36	11.9%	1.75 [1.17, 2.62]
You 2023	13	35	5	35	3.7%	2.60 [1.04, 6.52]
Zhao 2025	30	50	18	50	13.4%	1.67 [1.08, 2.57]
**Total (95% CI)**		**537**		**534**	**100.0%**	**1.78 [1.50, 2.10]**
Total events	240		134			

Heterogeneity: Chi² = 4.54, df = 10 (P = 0.92); I² = 0%
Test for overall effect: Z = 6.71 (P < 0.001)

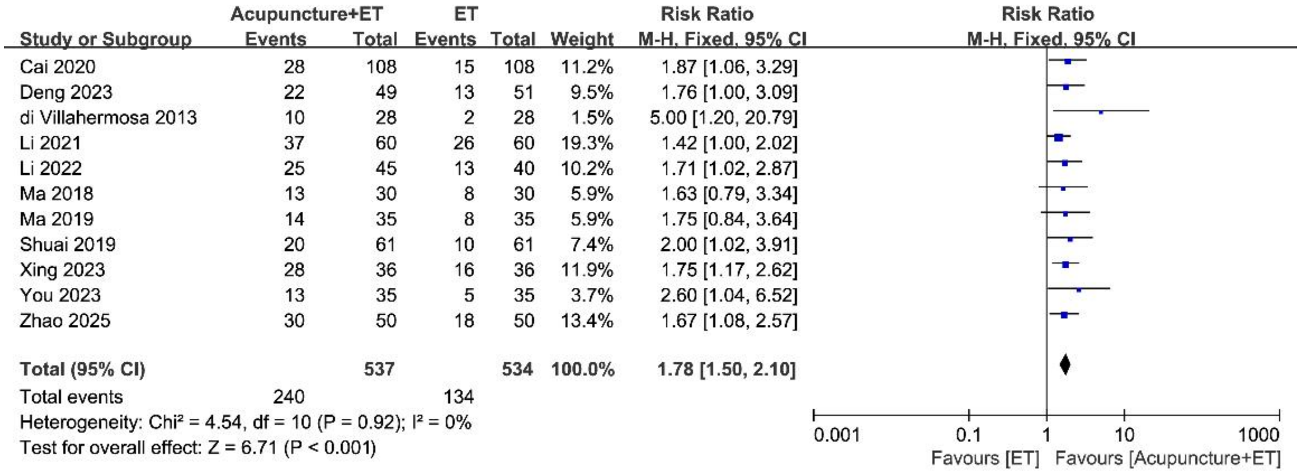

5 Appendix 5: GRADE Evidence Profile for All Outcomes

Certainty assessment							№ of patients		Effect		Certainty	Importance
№ of studies	Study design	Risk of bias	Inconsistency	Indirectness	Imprecision	Other considerations	[Acupuncture+ET]	[ET]	Relative (95% CI)	Absolute (95% CI)		
**Clinic pregnancy rate**												
14	randomised trials	serious	not serious	not serious	not serious	none	327/709 (46.1%)	191/719 (26.6%)	**RR 1.73** (1.51 to 1.99)	**194 more per 1,000** (from 135 more to 263 more)	⊕⊕⊕◯ Moderate	
**Endometrial thickness**												
11	randomised trials	serious	very serious	not serious	not serious	none	517	525	-	MD **1.2 higher** (0.75 higher to 1.66 higher)	⊕◯◯◯ Very low	
**Endometrial pattern**												
5	randomised trials	serious	very serious	not serious	serious	none	150/206 (72.8%)	153/206 (74.3%)	**RR 0.96** (0.73 to 1.27)	**30 fewer per 1,000** (from 201 fewer to 201 more)	⊕◯◯◯ Very low	
**Endometrial pulse index**												

Certainty assessment							№ of patients		Effect		Certainty	Importance
№ of studies	Study design	Risk of bias	Inconsistency	Indirectness	Imprecision	Other considerations	[Acupuncture+ET]	[ET]	Relative (95% CI)	Absolute (95% CI)		
5	randomised trials	serious	very serious	not serious	not serious	none	211	205	-	MD **0.49 lower** (0.93 lower to 0.06 lower)	⊕◯◯◯ Very low	
**Resistive index**												
6	randomised trials	serious	very serious	not serious	not serious	none	271	265	-	MD **0.21 lower** (0.3 lower to 0.13 lower)	⊕◯◯◯ Very low	
**Embryo implantation rate**												
3	randomised trials	serious	not serious	not serious	not serious	none	124/381 (32.5%)	76/400 (19.0%)	**RR 1.69** (1.34 to 2.13)	**131 more per 1,000** (from 65 more to 215 more)	⊕⊕⊕◯ Moderate	
**Embryo Transfer Rate**												
5	randomised trials	serious	not serious	not serious	not serious	none	120/319 (37.6%)	76/317 (24.0%)	**RR 1.57** (1.26 to 1.96)	**137 more per 1,000** (from 62 more to 230 more)	⊕⊕⊕◯ Moderate	
**Live birth rate**												

Certainty assessment							№ of patients		Effect		Certainty	Importance
**№ of studies**	**Study design**	**Risk of bias**	**Inconsistency**	**Indirectness**	**Imprecision**	**Other considerations**	**[Acupuncture+ET]**	**[ET]**	**Relative (95% CI)**	**Absolute (95% CI)**		
4	randomised trials	serious	serious	not serious	not serious	none	70/159 (44.0%)	30/153 (19.6%)	**RR 1.82** (1.16 to 2.86)	**161 more per 1,000** (from 31 more to 365 more)	⊕⊕◯◯ Low	
**Biochemical pregnancy rate**												
3	randomised trials	serious	not serious	not serious	serious	none	48/187 (25.7%)	42/199 (21.1%)	**RR 1.27** (0.91 to 1.78)	**57 more per 1,000** (from 19 fewer to 165 more)	⊕⊕◯◯ Low	
**miscarriage rate**												
3	randomised trials	serious	not serious	not serious	serious	none	7/121 (5.8%)	14/109 (12.8%)	**RR 0.40** (0.17 to 0.97)	**77 fewer per 1,000** (from 107 fewer to 4 fewer)	⊕⊕◯◯ Low	
**SAS1**												
3	randomised trials	serious	very serious	not serious	not serious	none	144	147	-	MD **5.89 lower** (10.93 lower to 0.84 lower)	⊕◯◯◯ Very low	
**SDS**												

Certainty assessment							№ of patients		Effect		Certainty	Importance
№ of studies	Study design	Risk of bias	Inconsistency	Indirectness	Imprecision	Other considerations	[Acupuncture+ET]	[ET]	Relative (95% CI)	Absolute (95% CI)		
3	randomised trials	serious	very serious	not serious	not serious	none	144	147	-	MD **6.83 lower** (11.27 lower to 2.4 lower)	⊕◯◯◯ Very low	

CI: confidence interval; MD: mean difference; RR: risk ratio
